# Post-Hoc Pattern-Oriented Testing and Tuning of an Existing Large Model: Lessons from the Field Vole

**DOI:** 10.1371/journal.pone.0045872

**Published:** 2012-09-25

**Authors:** Christopher J. Topping, Trine Dalkvist, Volker Grimm

**Affiliations:** 1 Department of Bioscience, Aarhus University, Rønde, Denmark; 2 Department of Environmental Social and Spatial Change, Roskilde University, Roskilde, Denmark; 3 Department of Ecological Modeling, Helmholtz Centre for Environmental Research – UFZ, Leipzig, Germany; University of Fribourg, Switzerland

## Abstract

Pattern-oriented modeling (POM) is a general strategy for modeling complex systems. In POM, multiple patterns observed at different scales and hierarchical levels are used to optimize model structure, to test and select sub-models of key processes, and for calibration. So far, POM has been used for developing new models and for models of low to moderate complexity. It remains unclear, though, whether the basic idea of POM to utilize multiple patterns, could also be used to test and possibly develop existing and established models of high complexity. Here, we use POM to test, calibrate, and further develop an existing agent-based model of the field vole (*Microtus agrestis*), which was developed and tested within the ALMaSS framework. This framework is complex because it includes a high-resolution representation of the landscape and its dynamics, of the individual’s behavior, and of the interaction between landscape and individual behavior. Results of fitting to the range of patterns chosen were generally very good, but the procedure required to achieve this was long and complicated. To obtain good correspondence between model and the real world it was often necessary to model the real world environment closely. We therefore conclude that post-hoc POM is a useful and viable way to test a highly complex simulation model, but also warn against the dangers of over-fitting to real world patterns that lack details in their explanatory driving factors. To overcome some of these obstacles we suggest the adoption of open-science and open-source approaches to ecological simulation modeling.

## Introduction

Pattern-oriented modeling (POM) refers to the multi-criteria design, selection, and calibration of models of complex systems [1]. The basic idea of POM corresponds to the overall strategy of science, i.e. to use observed patterns, which are characteristic of a certain system, for detecting the mechanisms that generate these patterns and therefore are likely to be key elements of the system’s internal organization [2]. For complex systems, single patterns are usually not sufficient to narrow down the range of possible generative mechanisms. Therefore, multiple patterns are used, which are observed at different scales and hierarchical levels. For example, cycles in the abundance of small mammals are a striking pattern, but usually do not contain enough information to unambiguously identify the mechanism which generates these cycles in reality. Additional patterns are needed, for example changes of cycle characteristics in response to weather, latitude, type of predators, etc., or changes in behavior in high- and low-density situations.

POM comprises three interrelated elements, which are briefly explained in the following (more detailed descriptions are in Grimm and Railsback [1] and Railsback and Grimm [3]). First, for complex systems multiple patterns should be used for model design, i.e. a model should not only include those factors which are considered essential for the model’s purpose, but also entities and processes that would allow patterns to emerge which are considered characteristic for the system’s structure and functioning. Such patterns can be taken from empirical observations and literature, from discussions with experts, and sometimes from existing theory. These patterns can be complex or simple, striking or relative weak, and possibly contain a lot of information or only a limited amount. Then, criteria are defined for deciding whether the model reproduces each pattern. Simple qualitative criteria should be used first, for example visual inspection of trends or whether or not average outputs are within confidence limits of observed data. The model is then revised until the most important patterns observed in reality also emerge in the model.

Second, patterns are used to contrast alternative sub-models representing a certain key process. For this, the alternative sub-models, for example of foraging, competition, or habitat selection, are implemented one at a time in the full model. Then, the alternatives are evaluated by testing how well the full model reproduces the set of characteristic patterns defined before. Sub-models that cannot reproduce one or more patterns are rejected. This is repeated until the best sub-model has been identified, which might require revising the original set of alternative sub-models or using additional patterns to enhance the ability to distinguish between sub-models.

Thirdly, multiple patterns can also be used for calibration of entire sets of unknown parameters. This works the same way as pattern-oriented model selection: each pattern is used as a criterion for acceptance but now it is parameter values being accepted or rejected. This approach is similar to “inverse modeling” or “Monte Carlo filtering” techniques used in other disciplines. It includes the following steps: identify parameters that need to be calibrated. These are particularly uncertain parameters, and those to which the model is particularly sensitive; identify calibration criteria, which often are categorical, i.e. it is checked whether a certain model output falls within a certain range. Model outputs should be observed in the same way as their real counterparts. Then, create a large number of parameter sets by varying the unknown parameters. Sampling techniques like Latin hypercube sampling can be used. Finally, run the model for all these parameter sets and discard those which ‘violate’ one or more of the test patterns. Use the ‘surviving’ parameter sets to run the model for addressing the model’s original questions.

POM is used implicitly by many experienced modelers, but it has been suggested that it be made an explicit strategy for utilizing observed patterns in a more systematic way [2]–[6]. The label “pattern-oriented modeling” is not common in the literature but the underlying concepts of simultaneous inverse modeling using multiple real-world and model data comparisons are increasingly used in ecology and other disciplines for developing models [1]. The resulting models are usually of moderate complexity with typically 10–20 parameters.

However, there are established and well-tested models of ecological systems which were developed without referring to POM and which are of high complexity, for example agent-based models of shorebirds [7], individual-based models of tropical rain forests [8], landscape succession models [9], or global vegetation models [10]. These models are complex because their ultimate purpose is prediction, accordingly they have to consider, e.g., multiple species, environmental drivers, heterogeneity in time and space and among individuals, local interactions, low-level processes like physiology, metabolism, or adaptive behavior, and stochasticity. Could POM also be used to maintain, test, and even develop such existing models? This would be highly desirable because testing complex models is hard, and even harder to communicate. POM could thus help to improve such models and facilitate their acceptance by decision makers by showing that they are able to correctly reproduce multiple patterns observed in reality.

We therefore decided to try to use POM to parameterize an existing complex model as well as to test the model’s ability to recreate the real world patterns chosen. While POM is usually aimed at building models of low to intermediate complexity, we use it here as a post-hoc application to a complex model.

The POM approach used generally follows the concepts developed by Latombe *et al* [11], in proposing a set of emergent patterns and a more qualitative assessment, and bearing in mind the intended domain of applicability. In this case the model chosen was required to replicate a wide range of behaviors and operate in a wide range of environmental conditions. As a consequence, patterns with a high level of emergence were assessed. The aim was thus to avoid constraining the flexibility of behavior of the final model by over-fitting, identified as a potential problem with simulation models by [12].

In contrast to established methods for design of simulation experiments, model calibration, and sensitivity analysis e.g. [13], [14] our approach is experimental and largely based on experience. We nevertheless believe it is important because standard methods for model analysis do not work for complex simulation models, so that specific pragmatic methods need to be developed. Many elements of our approach, however, should be useful for analyzing any complex simulation model. Moreover, we believe that virtually all developers of complex but predictive models have their own proven toolkit of tying their models to data. By explicitly describing and publishing our approach we hope to contribute to a culture of publishing these experimental methods, so that an appropriate methodology for tuning and improving complex simulation models will emerge from existing experience.

### The ALMaSS System and the Vole Model

Here, we use POM to test and develop an agent-based model of the field vole (*Microtus agrestis*), which was developed within the ALMaSS framework [15].

#### The ALMaSS system

ALMaSS couples mechanistic rule-based modeling of animal individuals (agents) with comprehensive inputs of environmental drivers and dynamic landscapes to create a flexible tool for evaluating scenarios that cannot be or should not be tested in real life, e.g. policy changes, farming changes, risk assessments [16]–[19], as well as more theoretical applications [20]–[22]. The system contains animal models that simulate the ecology and behavior of a range of species, including *M. agrestis*, at an individual level (agents), together with the agent’s interactions with conspecifics, predators and the environment.

The environment modeled in ALMaSS incorporates topography as a GIS map of habitat elements, and historical weather data. The map resolution is 1 m^2^ and the time-step for updating vegetation and management information in the landscape is one day. The structural landscape elements are divided into 40 different, extensible, classes including forests, buildings, roads and roadside verges, water features, hedges, field boundaries, and fields. These elements may have associated non-woody vegetation which will grow dependent upon climate and management, such as harvest or fertilizer application [23]. All agricultural managements are controlled by farmers who farm their virtual farms within the landscape. These farmers will apply crop rotations to their fields depending upon their farm type, which determine which crops are grown, in what order and with what area coverage. Management of these crops is via management plans which determine the order and timing of agricultural operations. These operations are again dependent upon probabilities, weather, and the history of past decisions. Other important human activities modeled include cutting of roadside verges in summer. The result is a realistic simulation of spatially and temporally located management events occurring in the landscape which are available as spatially-related information for the animal models which in turn can alter their behavior accordingly.

As an attempt to overcome the project life-time and size limitations to complex model development and provide better project accessibility, the ALMaSS system has been released as an “open science project” to provide: an opportunity for international collaboration in modeling over the internet; transparency in modeling and model testing; to facilitate the reproducibility of scientific results; freely available source and public availability and reusability of scientific data; and public accessibility and transparency of scientific communication.

This open project is in its infancy but the aim is to open the ALMaSS models to all interested participants and provides a long-term resource for bringing together data and models for iterative testing and improvement (see http://ccpforge.cse.rl.ac.uk/gf/project/almass/). Since this project is not contingent upon a single person or research funding, it is hoped that it could grow as a community based activity facilitating wider collaboration, model improvement, and access to data.

#### The vole model

*M. agrestis* is one of the most well studied small mammals with hundreds of studies covering molecular ecology e.g. [24], behavioral ecology e.g. [25], predation [26], [27], feeding ecology e.g. [28], [29], habitat selection e.g. [30], [31], and cyclic dynamics in particular e.g. [32], [33]. This, plus the fact that this species is widespread geographically with predictable habitat requirements, made it an ideal species for inclusion in ALMaSS.

The ALMaSS vole model was originally constructed in 1999–2002 and has since been used in a range of pure and applied studies e.g. [18], [19], [34]–[36]. Throughout development, the ALMaSS vole model has been subjected to plausibility tests, as well as ad hoc tests involving visual debugging [37] and internal validity and code testing [38]. However, until now the model has not been subjected to a formalized set of tests. Partly this is due to the fact that testing this type of model has generally been an *ad hoc* affair, if done at all, and partly because extensive testing of complex models is a time and data demanding process.

With a simple model framework it would be possible to attempt a statistical assessment of model testing. This might be based on AIC [39], [40], maximum likelihood [41], or Approximate Bayesian Statistics [42], [43]. However, for the vole model, the high number of parameters and long run-times make traditional statistical testing unfeasible. Simplification was also ruled out, because we wanted to keep the high flexibility and predictive power of the model and its framework. Thus, even though simplification might be possible for any given scenario, the cost would be a reduced predictive ability for other scenarios; the ultimate result being the need to develop and test multiple versions of the model for each new scenario.

The vole model has undergone a number of small changes since its original creation [15]; but here we adopt the version used by Dalkvist *et al* [19]. A full description of the model is not presented here, since a complete documentation of the model exists, using ODdox [44], which is a merger of a standard format for describing ABMs, ODD [45], and Doxygen, a software tool for directly generating documentations from computer programs [46]. This documentation is available at: http://www2.dmu.dk/ALMaSS/ODdox/Field_Vole/V1_02/index.html.

Since a version of the ALMaSS field vole model already existed prior to starting the POM procedure, it is useful to have a short description of the original form of the model. Deviations from this form are indicated as the results of the POM exercise.

#### A short description of the ALMaSS vole model prior to POM testing

The modeled field voles consisted of three life-stages, juveniles and adult females and males. During its life-cycle a vole could engage in a number of behaviors based on information obtained from its local environment and con-specifics. The vole entered the simulation at the location of its mother’s nest when it was weaned at day 14. It entered the simulation as either female or male, assuming an even sex ratio and started off by searching for a suitable territory. Voles could not deplete food resources in the landscape, preventing bottom-up regulation from food availability; however, density dependence was incorporated through local competition for territories.

Each day in the simulation the vole would start by assessing the local environment or its territory. Other behaviors could subsequently follow dependent on the information received during this process. A vole needed to have a territory in order to breed. A male could mate with a female if his territory overlapped her position. If this was the case for more than one male, she chose the one closest. Younger voles that found themselves in an older vole’s territory of the same gender with an overlap of more than 50% were forced to move. The criteria for assessing territory quality varied with the season and for the mature male during breeding season included the presence of mature females.

The breeding season typically started 5^th^ of April and ended 1^st^ of October. The start date was determined by the time at which new green grass growth occurred. This was under the control of vegetation models which were dependent upon the temperature. Currently, this is the only weather/vole interaction incorporated in the model. The length of the breeding season was varied by changing the end date to the 1^st^ of September or 1^st^ of November to simulate a short or long breeding season respectively. Mortality was modeled as being the result of predation, starvation, if they spent too much time in unsuitable habitat, or by reaching their physiological lifespan limit of 15±3 months. Mortality also included infanticide attempts if the mature male moved beyond the bounds of his original territory and encountered females with un-weaned young. His success would depend on the age of the young.

### POM Procedure

**Figure 1:**
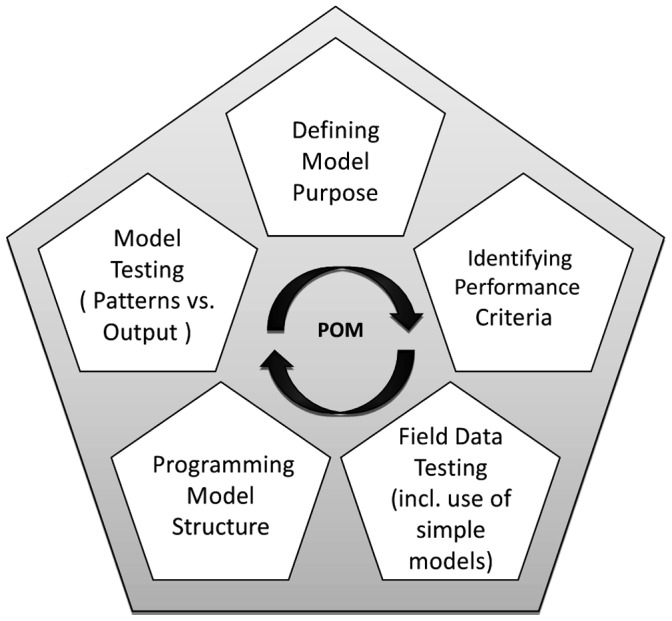
POM procedure for improving a model’s structure and parameterization. This is done by comparing model output to multiple patterns observed at different levels of organization and scales (from [44]). Field data testing is checking the internal consistency of the pattern data. Although not part of the traditional POM procedure this has been found to be a very necessary precaution due to errors and lack of detail in published data descriptions.

The POM approach used here follows Topping *et al* [44] and defines a number of real world data patterns to which the model output is compared. The process is iterative: after defining a model question it is necessary to traverse the complete POM process as depicted in Fig. 1, at least once. This process includes fitting parameter values as well as making structural code changes as necessary. Thereafter, should the model performance meet the performance criteria, the modeling cycle will be stopped, sensitivity analysis and documentation performed, and the model can be used for its intended purpose. In this iterative procedure, we focused on 17 parameters related to the vole sub-model of ALMaSS (see Table 1 in the Results section). Vole-unrelated parameters in the ALMaSS model framework remained untouched by this process. In some cases the vole parameters have been estimated by field studies and are available in the published literature. Since the precise values of these parameters (e.g. male territory size) are likely to be specific to a particular study or method, the published values were considered to be guidelines only. However, since the parameter values were not constrained in the model testing processes these parameters provide a useful secondary test of the model fit obtained, i.e. fitted values should fall close to the published values.

**Table 1:** Parameters varied (variables) as a result of model-cycle testing and the parameterization resulting from the POM testing.

Parameter Ref	Function	Value 2003/2009	Value after POM
V1	Male minimum reproductive age (days)	40	30
V2	Female minimum reproductive age (days)	20	23
V3	A multiplier to get a quality score from area (e.g. 1.5×minimum home range)	2.0	2.1
V4	Minimum female territory radius (m)	8	8
V5	Maximum female territory radius (m)	16	8
V6	Minimum male territory radius (m)	12	9
V7	Maximum male territory radius (m)	20	23
V8	Age difference needed before a male can ‘evict’ a younger male (days)	0	30
V9	Additional probability of mortality on dispersal	0	0.055
V10	Daily unattributed mortality probability	0.003	0.0025
V11**	The date in autumn at which reproduction cannot be started (day)	273	230
V12	The probability of moving if there are no females over-lapping a male’s territory	NA	0.0505
V13	Threshold number of voles in a territory for density dependence effects	1	4
V14	The temperature at which grass is assumed to grow (triggers breeding if achieved for 7 consecutive days) (°C)	5	3.552
V15	The date before which breeding is impossible regardless of temperature (day)	70	80
V16*	The number of consecutive days a vole can disperse without dying (days)	5	infinite
V17*	Probability of infanticide attempt	100%	100%

* V1–V15 were subsequently utilized in the sensitivity analysis, V16–V17 were found to be insensitive and therefore the effect of varying these was not reported.

** V11 is a climate dependent parameter, fitted to Finnish conditions from Pattern Set 1. It should be adjusted when applied to other regions.

#### Definition of model purpose

Here, the model’s purpose is to model the population and spatial dynamics of voles as accurately as possible. This model is intended for use in a range of scenario analyses for pesticide impacts, land-use changes and population dynamics studies, hence the aim was to obtain a broad range of realistic responses rather than fit a narrow set of conditions.

#### Choice of real world data patterns and modeling approach

Patterns were selected to be non-trivial emergent patterns, defined as ‘above comportment level 0′ by Latombe *et al* [11]. They were also selected to avoid redundancy (e.g. female density was used as well as sex ratios thus making male densities redundant). In order for real world data to be considered suitable as a data pattern for model comparison it also needed to fulfill two other basic criteria. Firstly it should be considered to be representative of the system modeled; secondly it must be possible to use ALMaSS to recreate similar conditions to those under which it was collected. After reviewing the available literature studies the following four sets of basic patterns were selected (see also supporting information ‘File S1 Pattern Set Data.xlsx’):

#### Pattern set 1: age and sex structure of the population

Myllymaki [47] carried out a study in southern Finland in 1968 in which age and sex structure of the population was monitored from May to September using live-trapping in a population fluctuating with a four-year cycle. At the time of sampling the population was in its increase phase. Voles were trapped in areas of activity identified in the spring and the result is a detailed, but non-spatial, picture of the population structure. From this data five patterns were identified as suitable for fitting:

P1.1 Sex ratio on day 90 (1∶1 M:F). Fitting criterion ±2%.

P1.2 Sex ratio on day 200 (1∶1.95 M:F). Fitting criterion ±2%.

P1.3 Mean breeding season female density (75 Ha−1). Fitting criterion ±2%.

P1.4 Male age structure with season. Fitting criterion - least squares difference combined with other patterns and this overall measure minimized.

P1.5 Female age structure with season. Fitting criterion - least squares difference combined with other patterns and this overall measure minimized.

The simulation approach for P1.1–P1.5 was to simulate a population of voles living in a block of high quality habitat surrounded by an equally large area of dispersal-only habitat. No predators were included since the population was in its increase phase in 1968 and hence specialist predation would be at its lowest. Each simulation was run for 20 years, but for evaluation the first 10 years were discarded to avoid including possible effects of initial conditions. In order to adjust for the differing climate regime in Finland the starting/stopping conditions for breeding were allowed to vary and were included in the set of parameters for fitting. After each run, mean sex-ratios on days 90 and 200 (±15 days) were calculated, as was female population density at day 200 (±15 days). Deviation from the target pattern was recorded for each simulation run.

Population structure in the middle of each month of May-September was recorded and converted to a proportion. The squared difference as a mean across all five months was used to compare goodness of fit for both males and females to patterns P1.4 and P1.5.

#### Pattern set 2: vole densities across multiple habitat types

The literature was searched for densities of *M. agrestis* for non-cyclic populations (Table 2). In cases where the data was pooled with other species, or where a clear description of the habitat was missing, or where the field voles where sampled in a habitat type not represented in the model, the data was discarded. In cases where the data was presented as field voles/100 trap nights or catch per removal quadrat, we used the methods of Wheeler [48] and Hansson [49], respectively, to convert the measure into voles/ha. Densities from the literature were log_10_-transformed to normalize them and calculated as a mean within each of the seasons; spring (March, April, May), summer (June, July, August) autumn (September, October, November) and winter (December, January, February) and listed together with their standard deviations. Due to limitations to the method of Wheeler [48], densities of less than 9 voles Ha^−1^ were lumped as a categorical variable. Weather data used as input came from 1990–1999 from a weather station in Central Jutland (UTM 32-ED50∶543, 6,244) and comprised daily temperature and precipitation.

**Table 2:** Literature used to obtain density estimates for comparison to model outputs.

Reference	Habitat	Measured	Recalculated to voles/100 trap nights
[68]	Set-aside	Voles/100 m transect	Yes [48]
[68]	Unmanaged grassland	Voles/100 m transect	Yes [48]
[68]	Linear features	Voles/100 m transect	Yes [48]
[68]	Pasture tussocky	Voles/100 m transect	Yes [48]
[68]	Pasture low yield	Voles/100 m transect	Yes [48]
[68]	Woodland	Voles/100 m transect	Yes [48]
[68]	Field crop	Voles/100 m transect	Yes [48]
[48]	Unmanaged grassland	Density	No
[69]	Unmanaged grassland	Density	No
[69]	Pasture tussocky	Density	No
[70]	Unmanaged grassland	Density	No
[71]	Unmanaged grassland	Density	No
[72]	Unmanaged grassland	Density	No
[73]	Unmanaged grassland	Voles/100 trap nights	Yes [48]
Olsen pers. comm.	Unmanaged grassland	Voles/100 trap nights	Yes [48]
Olsen pers. comm.	Linear features	Voles/100 trap nights	Yes [48]
Olsen pers. comm.	Field crop	Voles/100 trap nights	Yes [48]
[74]	Forest plantation	Catch/SQ	Yes [49]
[59]	Pasture tussocky	Catch/SQ	Yes [49]
[75], [76]	Pasture tussocky	Density	No
[75], [76]	Pasture low yield	Density	No
[77]	Woodland	Voles/100 trap nights	Yes [48]

**Figure 2:**
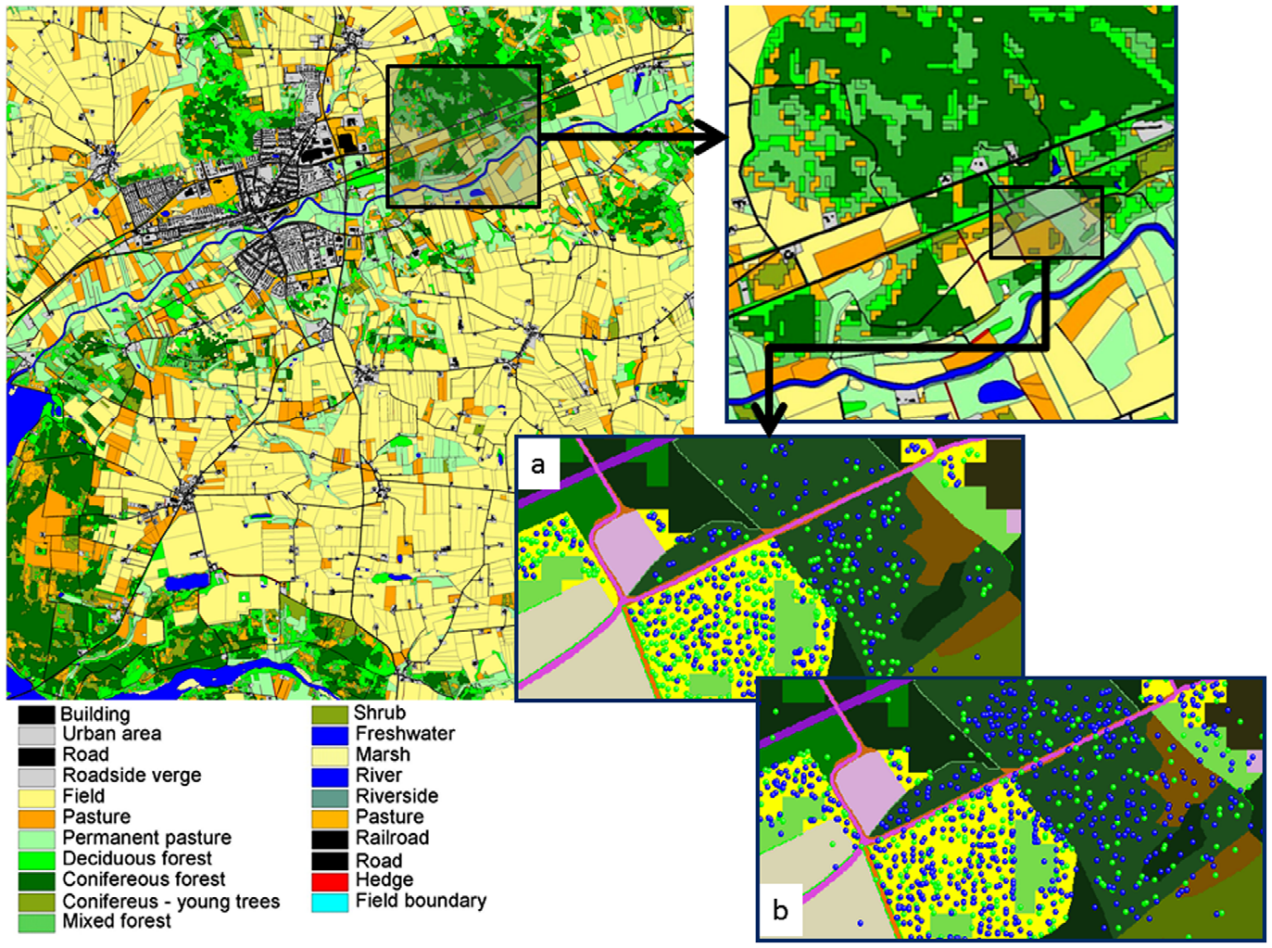
The GIS map of the 10×10 km landscape was used as the landscape for pattern set 2 testing. Exploded panels show the detail of the map and screenshot of the ALMaSS vole model shows a) male and female locations (blue & green dots) in February, b) June locations. Note some habitats have been recolonized or are occupied by dispersers in June.

Simulations were constructed using a typical Danish landscape (Fig. 2). Each parameter configuration tested was simulated for 30 years, with the first 20 years of data discarded. Weather inputs were for the years 1990–1999 from the area mapped. Mean densities were calculated for all occupied habitat patches in the map for each of the four seasons. Patches were considered only if they were >1 ha in area. The exception to this was made for unmanaged grassland and linear features (e.g. hedges), in which case a lower limit of 1000 m^2^ was used, since these features were rarely >1 ha in size. This prevented chance events of tiny patches containing one vole from biasing the results. All in all there were 22 observations resulting from the combination of literature studies, habitat and season. Deviation from these patterns was assessed as the total absolute deviation on a natural log scale per habitat and season. To provide a restrictive test an arbitrary pass mark of 10.0 was used to assess whether the fit was acceptable (which corresponds to a mean of <0.5 [22 observations] on a log*_e_* scale).

#### Pattern set 3: dispersal

Field vole dispersal was studied in southern Sweden by Sandell *et al*. [50], [51] in a homogenous wet meadow using three 14×7 grids of live-traps with 7 m between traps, and 30 m between grids. Four main results were selected as patterns for matching and criteria for fit were defined as:

P3.1– Strong adult philopatry. Dispersal was only greater than two home-range diameters (males 90 m, females 70 m) for <2% for both males and females. Pattern fitted when both measures are less than 2%.

P3.2– Mean distance moved between trapping was 10.2 m (±11.1 m) and 9.0 m (±10.2 m), males and females respectively. Pattern fitted when both measure lie within confidence limits.

P3.3– Mean maximum movement distances per individual were greater in males than females (28.6 m ±19.0 m cf 22.4 m ±16.3 m). Pattern fitted when both measures lie within confidence limits.

P3.4– Natal dispersal distances were high. Sandell et al. [50] found 13.8% of natal dispersal to be over 2 home-ranges, and 60% within 1 home range. Pattern fitted when both figures are matched to within ±5 & 10% respectively.

To simulate this study a homogenous area of grassland 500 m×400 m was simulated as being surrounded by forest. Three grids of pitfall traps were simulated in the center of this area and spaced as in the original study (see above). The simulation was run for 10 years to allow the population to equilibrate. Following this the simulation was run for a further two simulation years and any vole within 1 meter of the trap location was identified on a daily basis. The trap location, natal location, date, unique identification number, age, and sex of the vole were recorded.

Using the identification number to track voles in the same way as the mark-release-recapture was done in the real study it was possible to recreate the statistics provided by the original studies. Natal dispersal measurements were, however, restricted to voles born within the grid plus one home-range diameter to simulate the same conditions as the original study.

#### Pattern set 4: the ability of the model to create realistic predator-prey cycles

**Figure 3:**
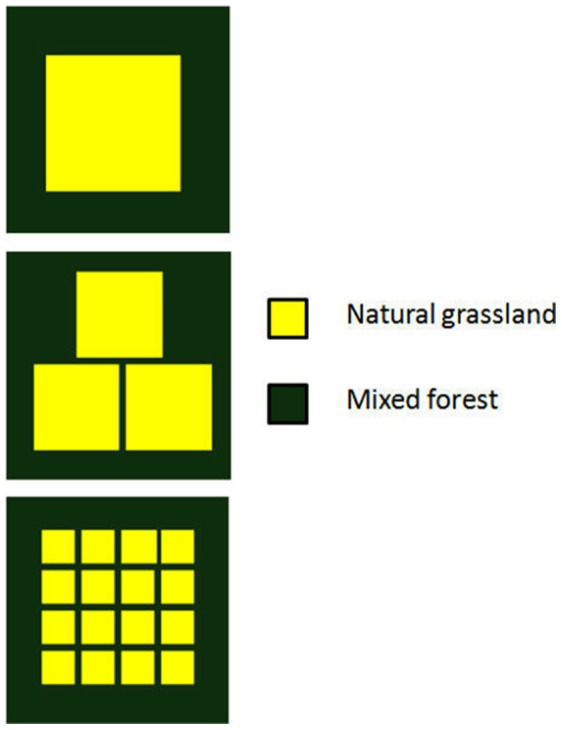
Three simplified landscapes used for testing the model’s ability to produce vole population cycling. To test for the emergence of cycles, predator characteristics were varied in conjunction with these landscape structures.

Vole multi-annual cycles is one of the best known population patterns in ecology [32], [52]–[56]. It was therefore considered important that the vole model could simulate these cycles as emergent properties of predator-prey and landscape structural interactions. Two types of cycling and non-stable fluctuations could be identified from the literature. The ability of the model to create these cycles by varying predator numerical response and landscape structure was therefore tested. To pass the test, the model had to produce 3 types of fluctuations; 1) stable 5 year multi-annual fluctuations with amplitudes of around 3 (calculated as log_e_(max N/min N)), and a low phase of 2–4 years in between; 2) less stable fluctuations with cycle length of 3–5 years with lower amplitude (∼2); 3) non-stable fluctuations with low amplitudes (∼1). Landscapes used for this test were structurally simple (Fig. 3). Except for numerical response the predator behavior was kept constant for each test. Specialist predators were assumed which were characterized by a delayed numerical response to changes in prey density. These were modeled to require a relatively high number of voles in order to survive and a low number of voles to reproduce. Predator dispersal would occur within a few days of unsuccessful hunting. Their home range and dispersal ability was relatively low in order to represent small mammalian predators [22].

## Results

Fitting the parameters and traversing the model cycle required approximately 48,000 simulation runs. Stochasticity resulting from decision processes in the model was reflected in the patterns [57], hence, a minimum of ten replicates of any parameterization was required during fitting to avoid using erroneous signal information causing divergence from the best parameterization.

In the following, we first provide a summary description of the changes in model structure and parameters that resulted from the pattern-oriented calibration. Then, for each of the four pattern sets, we describe in more detail the insights gained from the process of model fitting. Finally, results of the sensitivity analysis are presented.

### Results of Applying the POM Cycle

Initial and final values of the 17 calibration parameters are presented in Table 1. In addition, a number of structural changes were made to the model structure to improve model behavior. It is important to note that the POM process is not just a re-calibration, but can, and did, also result in changes to the underlying model. These changes were:

- Introduction of an age difference requirement before an older male could evict a younger male from a territory (V8; V-references refer to corresponding variables in Table 1).
- The introduction of an additional mortality factor when voles were dispersing as a probability of dispersal mortality per dispersal event (V9).
- Introduction of a variable threshold number of voles, scaled to sex-specific minimum territory size, below which density-dependent effects were ignored. This was measured as a local number of voles present within the bounds of the vole’s territory. This addition altered the territory quality assessment method compared to previous versions (V14).
- Allowing variability in the minimum reproductive age. This was found to be necessary to provide a fit to the age structure, which had previously been fixed at literature values.
- A restructuring of the code to allow the introduction of juvenile male and juvenile female classes needed for the age-structure outputs (Pattern set 1). This did not affect code function but did increase code readability and was necessary to obtain the differentiation of age classes in the model outputs.
- Inclusion of variable habitat quality based on digestibility already incorporated in the ALMaSS system [44]. Digestibility was given as 0.7 plus the square root of the proportion of new green biomass (<14 days old) out of total biomass, with a ceiling of 1.0. This allowed a 30% variation in habitat quality between fresh new growth and mature biomass. Further refinement of this feature was not undertaken due to a lack of suitable real world data for this species.
- Removal of starvation days as a concept (V16). This was found to be redundant after inclusion of V8 in ‘ii’ above since the additional dispersal mortality was more restrictive and on the face of it more realistic than a simple starvation threshold.
- Infanticide probability (V17) was also found to be insensitive, but this was retained in the model because this factor is a known feature of the ecology of this species and because it was considered that other scenarios (e.g. genetic or dispersal in low density populations) may require this feature to be enabled.
- Code was added to simulate live-traps and to produce output tailored for density and age-structure analyses. Although not affecting the model behavior, this code was necessary to produce the outputs needed for the dispersal evaluation (Pattern set 3 below).

During the fitting process a number of observations were made about the process as well as the final fit for each pattern set. These are detailed for each pattern set below.

#### Pattern sets 1 (P1.1–1.5): age structure and density

One important result was the inability to combine the results of the simulation approach to age structure with density measurements in large-scale landscapes. It was quite possible to obtain very good fits to Myllymaki [47] data, but these fits resulted in completely unacceptable fits to patterns of density across multiple habitat types (pattern set 2 patterns). Incorrectly set dispersal mortality parameters were identified as being the major cause of the discrepancy, and as a consequence it was decided to attempt to recreate a landscape structure similar to that sampled by Myllymaki in the Ahtiala study area, and refit the parameters.

**Figure 4:**
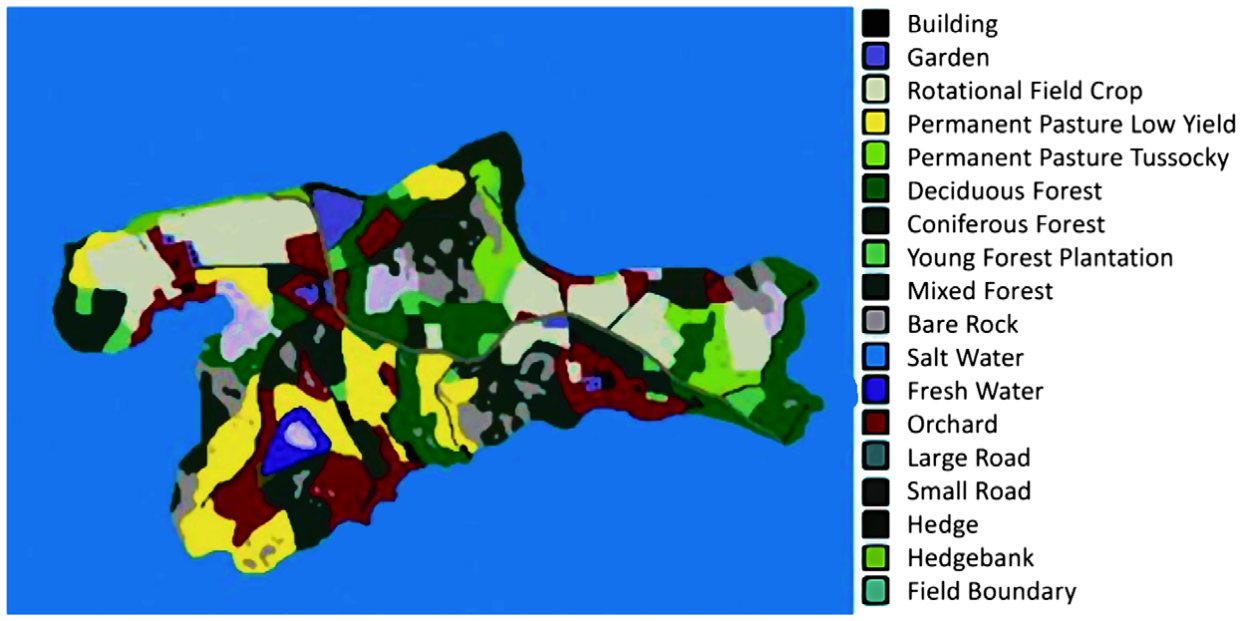
GIS map of the island comprising the Ahtiala study area from which the real world data was obtained to test model vole sex ratios and population age-structure.

The landscape was created by identification of the island of the study and subsequent mapping based on imagery from Google Earth. A number of the habitats could be identified from tourist route descriptions of old woodlands and orchards, and due to the topography many landscape structures will have remained constant since 1968 (e.g. rocky outcrops). The rest of the habitat patches had to be assumed to be as they were in the original study. Farming was considered to be cattle farms with pasture and crops of cereals and fodder beet. The resultant map (Fig. 4) was incorporated into ALMaSS and the model cycle re-started. Since the original study only sampled from high vole density areas and pooled all data, the same procedure was followed in ALMaSS. Hence, all vole populations in old orchards were counted, and densities were calculated as female voles per hectare orchard.

**Figure 5:**
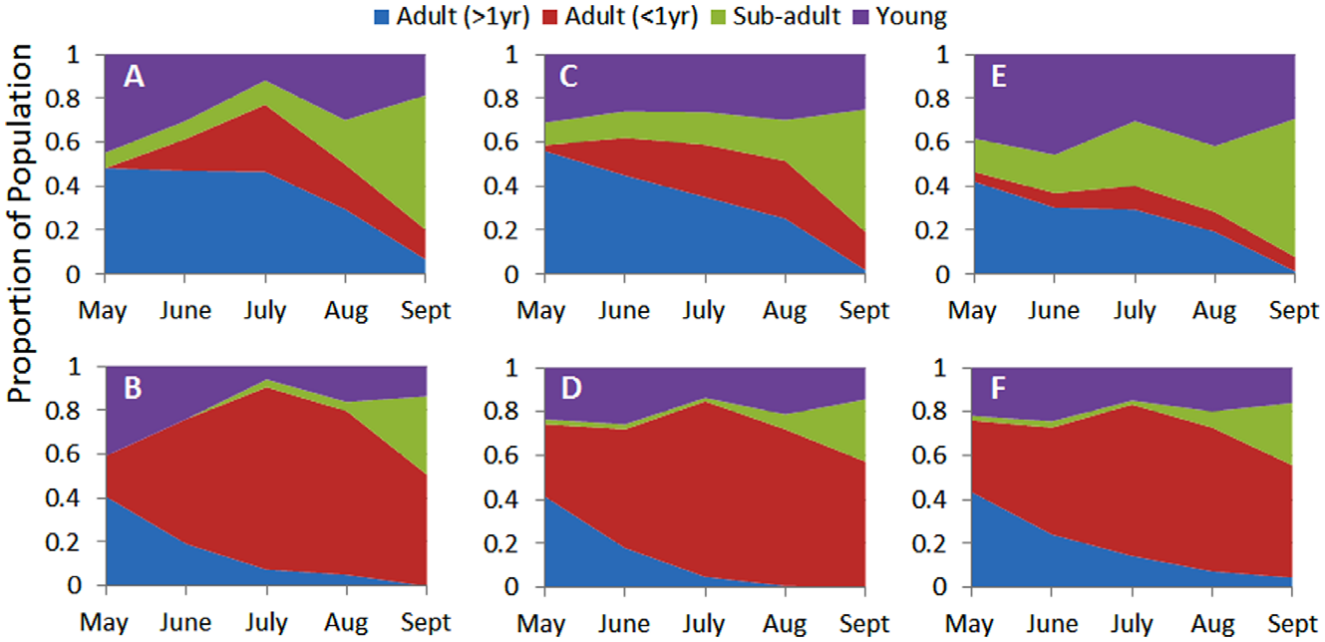
Age structure for males and females based on Myllymaki (1977) and the best fit model simulations, and final fit resulting from the POM exercise (accepted fit). A) Actual male age structure; C) Best fit model male age structure; E) Accepted fit model male age structure; B) Actual female age structure; D) Best fit model female age structure; F) Accepted fit model female age structure.

As the first stage in the testing cycle, based on the more realistic map of Ahtiala sample site, both male and female age-structures could be re-created with a high precision (Fig. 5). The best fit measurement for males and females was 0.088 and 0.075 respectively (Fig. 5 C & D). However, the procedure used to fit multiple patterns (pattern sets 2–4) resulted in sacrificing male and female fit somewhat to obtain the optimal fits to density and sex ratio patterns with fits of 0.298 and 0.121 (Fig. 5 E & F) respectively. Accepted deviation from fit for mean female density was +0.4%, sex ratio day 90 was +2.1%, and sex ratio day 200 was −1.3%.

As a secondary test of the model it is worth considering the fits of parameters for which we believe we know the approximate ranges. V1, V2, V4–V7 (Table 1) represent minimum reproductive age and territory size parameters. These were allowed to vary for fitting but we have good indications of expected values from the literature. In all cases the resulting fitted value matches the range of values reported from the literature well. In the case of minimum male reproductive age, this deviates by 6 days from the reported value [58], but this study did not look for earlier maturation, so it can only be considered a guide.

#### Pattern set 2 (P2.1): vole densities in multiple habitats

**Figure 6:**
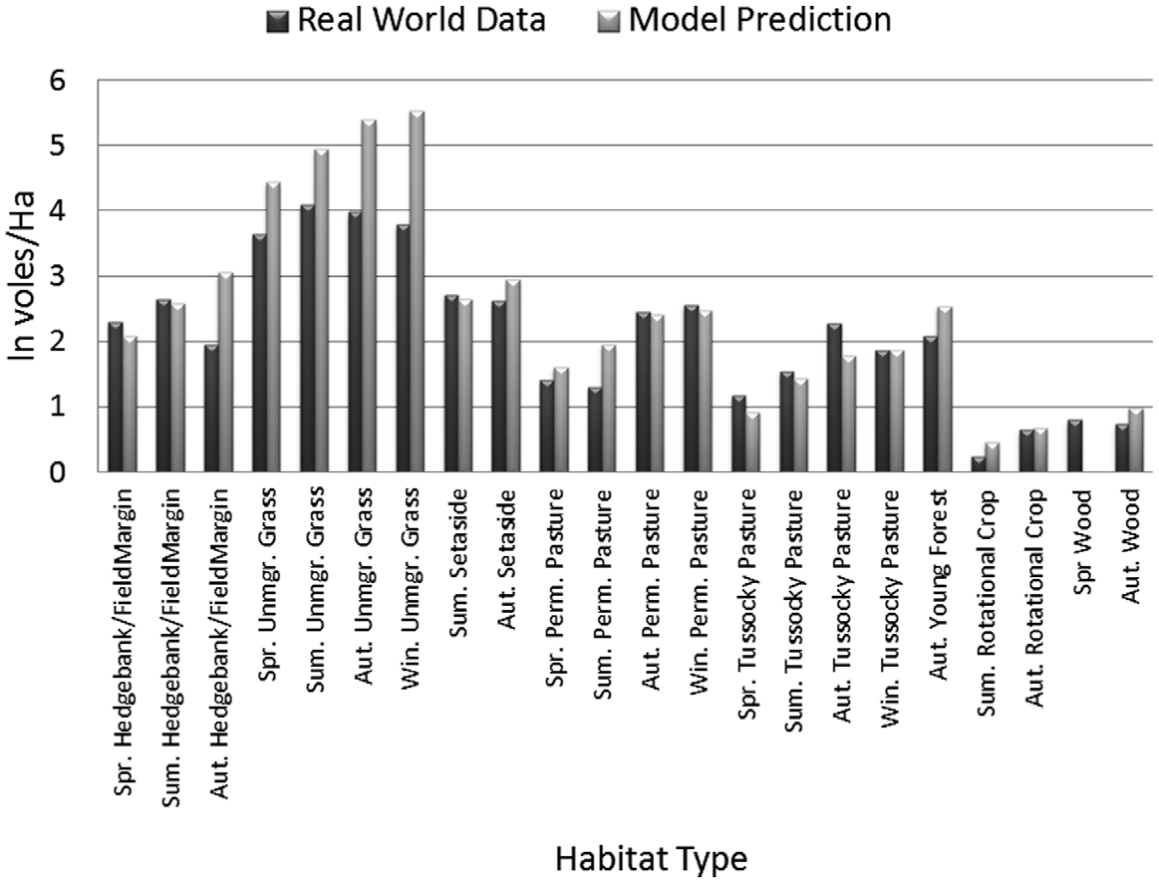
Real world means and model means for total vole density for a range of Danish habitats and sampling periods. X-axis abbreviations: Spring (Spr.), Summer (Sum.), Autumn (Aut.), Winter (Win.), Unmanaged (Umgr.) Permanent (Perm.).

Using this configuration for fitting to the heterogeneous landscape provided a mean absolute fit deviation across all habitats and dates of 0.4 (ln scale). The pattern of fits shows that with the exception of unmanaged grass areas there was no obvious bias for over or under estimating vole densities (Fig. 6).

#### Pattern set 3 (P3.1–3.4): vole dispersal

The model configuration found was capable of satisfying all pattern fitting criteria 3.1–3.4 (Table 3). Similarly to patterns from pattern sets 1–2, the dispersal fits were also found to be highly dependent on the precise simulation conditions. For instance, in ALMaSS it is straightforward to create natal dispersal statistics for the whole population. This however was not how it was done in the field study, where natal dispersal was assessed from data collected from the grids. Hence, it was important to restrict the assessment of data in the model test, disregarding any voles born further than one territory diameter from the grid area. In this case it was clear from the original research description what should be simulated, but grassland or other boundary conditions also affected the fits as did the conditions for assuming trap-captures. Increasing the area of trap influence led to decreased maximum distances moved as it became almost impossible for voles not to be caught in traps. Hence, more precise fitting was not considered desirable without better descriptions of the actual study area and conditions.

**Table 3:** The final model configuration simulation results for patterns 3.1–3.4 compared to those observed by Sandell *et al.* [50], [51].

Pattern Set 3 Pattern	Sandell et al	Model
Adult Male Philopatry (%)	1.4*	1.0
Adult Female Philopatry (%)		0.3
Mean Max. Male Dispersal (m)	28.6	41.9
Mean Max. Female Dispersal (m)	22.4	22.2
Mean Female Inter-trap Distance(m)	9	8.6
Mean Male Inter-trap Distance (m)	10.2	11.8
Mean Natal Dispersal Distance (m)	13.8	13.0
Natal Dispersal <2 Home-ranges (%)	61.0	70.1

* Only pooled sex data provided.

#### Pattern set 4 (P4.1): vole population cycling

**Figure 7:**
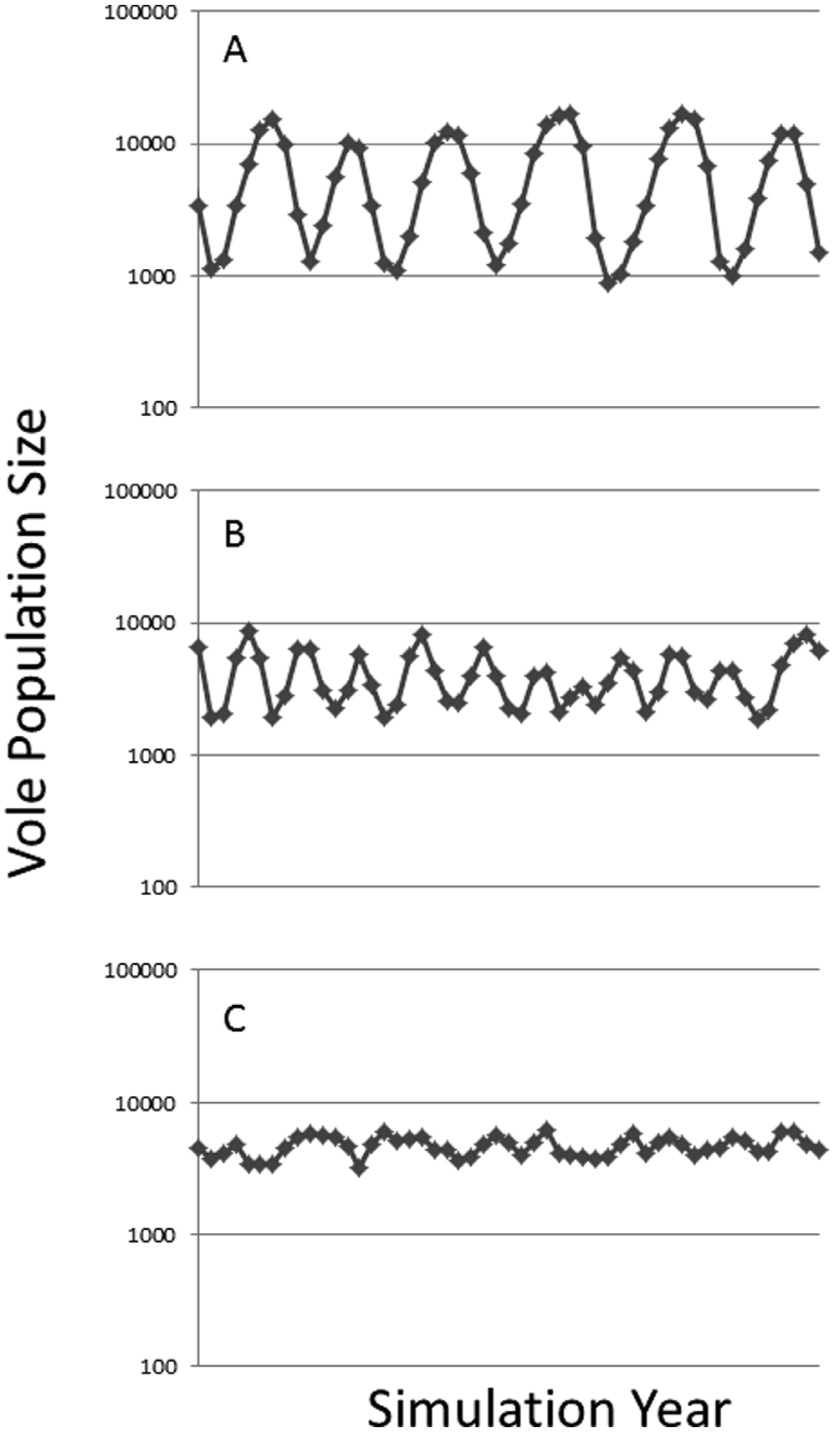
Three examples of 50 years of simulation using the parameterized model on three different landscapes (see Fig. 3) A) 1 patch; B) three patches; C) 16 patches.

The final model configuration was able to satisfy the criteria for stable multiannual cycles and non-stable population fluctuations. Similarly to the other patterns evaluated, population cycles were found to be highly dependent on landscape structure as well as predator configuration. Increasing the level of heterogeneity generally produced less stable cycles with lower amplitude whereas increasing the predators numerical response to vole density generated more stable fluctuations with high amplitudes (Fig. 7).

### Sensitivity Analysis

**Figure 8:**
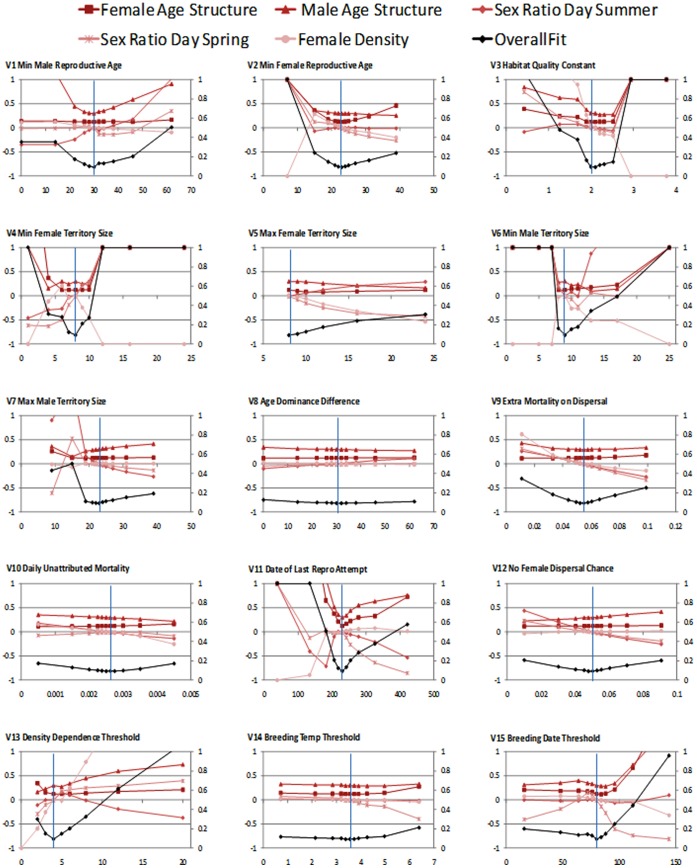
Graphs of sensitivity analysis for the 15 parameters tested. Fits to density, sex ratios and age structure are shown as proportion deviation from target pattern. X-axis denotes the parameter values used in each case, and the vertical line the actual parameter value chosen following POM testing (see Table 1). Overall measure of fit (black line) is the mean deviance and is capped at 1.0. All graphs are scaled to ±1.0 for proportion deviance from real world patterns (left y-axis), and 0–1.0 for measure of fit (zero being perfect fit) (right y-axis).

Following fitting of the pattern sets 1–4, sensitivity analysis is a natural progression, the main results of which are summarized in Fig. 8, for pattern set 1. The model was sensitive to a number of parameters, with V1–4, V6, V7, V11, V13, and V15 all causing more variable responses (±100% change in at least one response variable) at ±80% of their fitted value. Of these V1, V4, V6 & V7 all represent parameters for which we believe the values chosen lie within acceptable ranges. V2 is a model construct, essentially a scaling factor relating habitat scores to final quality and can therefore never be validated. V11 and V15 are both dates controlling start and finish of reproduction. Since this is thought to be primarily controlled by photoperiod [58], these dates are likely to also be reasonably accurate in that they result in sensible within season population dynamics. The model was only slightly sensitive to mortality factors (V9 & V10), and the chance of dispersal by males if there are no females present during the breeding season (V12).

Although not part of the sensitivity analysis *per se*, the iterative process of fitting to pattern sets 2–4 revealed further aspects of sensitivity. Pattern set 2 fitting restricted the parameter sets with respect to mortality, especially dispersal mortality parameters. Likewise pattern set 3 (dispersal) patterns further restricted both parameters related to dispersal and territory size. The vole cycles were also highly sensitive to input settings, although not parameters formally tested here. As thought to be the case in the real world, the emergent population cycles were dependent upon the landscape structure, predator specificity, and less so on vole parameter settings [22].

### Qualitative Changes in Model Behavior

The new post-POM version of the model still has an underlying structure very similar to the previous version. However, both model structure and parameter values have been altered, and subsequent usage of the post-POM model highlights three important differences compared to the pre-POM version. These are 1) a difference in relative densities in different habitats, often leading to much higher (2×) densities in optimal habitats; 2) a change in phenology with higher late summer numbers than previously and earlier cessation of breeding; 3) increased within-year dynamics in sex ratios. A number of toxicological impact assessments, however, remained largely unaffected e.g. [19]. The implication being that studies relying on changes in habitat structure or requiring analysis of population structures will be most affected by use of the new version.

## Discussion

The ALMaSS vole model was, like probably most complex models which are designed for making robust predictions, implicitly designed to simultaneously reproduce multiple patterns. Therefore we applied the POM approach to an existing model, both to explicitly check how well multiple patterns can be reproduced after calibration, and to see how the procedure could help improve model structure.

The final fit between model outputs and real world patterns was generally very good. The model was able to predict relative densities in a wide range of habitats and seasons, simulate within season population dynamics, natal and adult dispersal, and vole cycling. This indicates a high level of structural realism of the model. But it is important to keep in mind than even the best model still is a model, which means it still ignores more features of reality than it can include. Hence, we first discuss remaining issues of model performance which indicate limitations in the model’s structural realism. Only then do we discuss the pros and cons of our post-hoc POM approach to calibrating and complex models and improving and proving their structural realism.

### Limitations of Fits to Real World Patterns

There are four main weaknesses in the observed fits that merit discussion. The first is the generally high density prediction for natural grass areas compared to real world measurements. This was a consequence of the fit to pattern set 1 pattern set, i.e. mean female density of 75 voles Ha^−1^ at the peak of the breeding season in the Ahtiala landscape. Whilst there is little doubt that this was the case [47], the model assumes that all such areas are of equal quality to those found in Finland. This is clearly erroneous, and hence estimates of vole density are generally high. Clearly differentiation of unmanaged grassland is a feature that should be considered in future releases.

The second issue relates to changes in habitat quality. At least one factor thought to be important in shaping vole densities is not incorporated in the model, i.e. drought. Loss of high quality green food in summer has been reported to dramatically affect vole numbers [59]. The current model does allow for some variation in quality as a result of the green/dry matter ratio of vegetation, but this ratio is not yet altered by drought. Therefore, to improve the vole model it will be necessary to consider significant improvements to the ALMaSS unmanaged vegetation models.

The third point concerns the within-season changes in density. In Myllymaki [47] there was a clear decline in density in later summer, but this was not generally the case in the data from other studies. Three drivers may have caused this: externally caused increase in mortality (increased predation); internal birth and death processes (disease, early cessation of breeding); and/or changes in habitat quality (e.g. drought above). Externally altered mortality can be included by altering predator settings from the general background mortality to the coupled dynamics used to recreate the vole cycles. This does, however, require information about the prevalence of predator and their specificity. This is out of scope for this paper, but may well have been an issue affecting the differential fits between Finnish data source [47] and the predominantly Danish based density studies.

The fourth point is that our manual fitting method does not provide a description of the entire possible parameter space that could provide suitable fits. The sequential nature of our iterative fitting procedure means that fitting to a local optimum cannot be ruled out. Naturally a Monte-Carlo approach would provide a solution to this but would be logistically impossible due to the dimensionality of the problem and the need for separate scenario inputs for each pattern set. An alternative approach might be to use Approximate Bayesian Computation [42], [60] which might both help in automation of the parameterization, and to describe the resulting suitable parameter space. However, these techniques are as yet untried on models as complex as the ALMaSS vole model, and currently suffer from the long computation times needed per replicate.

A more general point arises from the example of infanticide. This behavior was found not to affect the fit to the observed patterns, but was retained in the model nonetheless. The argument following the common modeling practice would be that it should be removed as unnecessary complexity. However, this is a behavior that we believe to be part of the normal vole ecology [61], [62] and in certain circumstances (e.g. low density populations, genetic studies), it can be important. Since the purpose of this model requires it to have a wide domain of applicability, we judged that removal of this process on the grounds that the literature patterns we used do not support it, was not justified. In this case the model is a better representation of reality than the test data currently available and should not be constrained by this. An alternate way to view this is that the fact that infanticide happens could be considered a pattern; the result of the POM test is then obvious.

### Evaluation of the POM Exercise

As the model cycle was applied to the vole model it became apparent that the most difficult aspects of the fitting process were: 1) That the real world patterns were based on studies, the details of which were not adequately described for simulation purposes; 2) That the precise fit to the patterns was very much dependent upon the precise simulation inputs; 3) The patterns although not redundant were not independent either, nor should they be.

The first issue, inadequate empirical patterns, must be considered a general problem when testing detailed models on published studies. These studies were not conducted for POM testing; they are often old, being carried out in a time when focus was more on large long-term data sets rather than detailed descriptions of context. Pooling of sample data, inconsistent definitions, especially of habitat types, and uncertainty about the reliability of densities based on live-trap methods all contributed to the difficulties of interpreting and using old non-specific POM studies in this and previous ALMaSS POM exercises [44], [63]. To some extent some of this bias can be compensated for by altering the perspective for model sampling. For instance, the live-trap simulation approach used for the pattern set 3 dispersal patterns is an example of the “virtual ecologist” approach [64], [65], as is the restriction of sampling from high quality habitats on Ahtiala as carried out by Myllymaki [47]. Many other idiosyncrasies of pattern data cannot be dealt with in this manner and go undetected as stochasticity, e.g. the decrease in density in late season as discussed above.

The second issue, sensitivity of output to simulation input, is both a positive and negative feature of the ALMaSS vole model. The positive aspect is that the model exhibits behavior in response to changes in sensible inputs (e.g. landscape structure). This is clearly needed if we want to use to the model to evaluate factors such as change in land-use and management e.g. [18], [66]. The negative aspects are a result of the requirement for specificity in inputs, and the aforementioned problem of inadequate real world descriptions. In the case of the structure of the landscape from which our pattern set 1 data patterns originated, it was clear that assuming a too simple structure for fitting these patterns made it impossible to fit to the pattern set 2 patterns. This is precisely the idea of incorporating a number of patterns, i.e. to reduce the potential parameter space, but it also raises concerns of uncertainty in the real world patterns (over-)influencing the final model. In hindsight the map we constructed represented more realistically the structure of the study area than a homogenous block, but its precise details were probably inaccurate. Hence we have an unquantifiable uncertainty not in the model or parameters, but in the data we use to test the model. This phenomenon might be considered a passive over-fitting of the model and restriction of the effective domain of applicability.

The third issue, that patterns are not independent, can be considered a strength of the patterns selected here. Although as Latombe *et al*. [11] state, redundancy does not contribute to validating the parameterization, the fact that the patterns are not wholly independent is very important in limiting the potential parameter space. Considering the alternative where patterns are completely independent then it may be possible to adjust each output signal to the corresponding pattern by manipulation of independent variables. This would not improve confidence in the model, although the fit may be excellent! This could be considered to be analogous to an imposed response (sensu [67]), but at the level of the whole model rather than individual responses. Patterns observed in real systems are to be expected to be linked to each other, if they indeed, as we assume, all reflect certain aspects of the same overall internal organization of the real system. For example, sex ratio, emergent sex-specific mortality, and age structure all reflect the same processes, but emphasize different aspects. Getting these interconnected patterns right simultaneously is thus more of an insurance, or confirmation, that the model is on the right track, than mere redundancy. Nevertheless, it is important to include patterns from different scales and levels of organization.

Given the above considerations we would conclude that the ALMaSS vole model in its current, post POM form, is able to mimic many more or less independent patterns, many of them observed at highly integrated levels. Thus we assume that the model captures the internal mechanism of vole population dynamics in heterogeneous and dynamic landscape sufficiently well for the model’s intended purpose. The model is capable of a range of realistic behaviors, and does not appear to have obvious major flaws in matching the published vole study data. This does not mean, however, that we consider the model even close to perfect, but there is probably no vole-landscape model that has higher structural realism.

To conclude, we found post-hoc testing and tuning of a complex model a highly useful approach. Due to data constraints and lack of previously developed standard protocols, many steps in our approach required experimental decisions; being explicit about these decisions and about the structural realism that can be achieved with a model is, however, an important step towards transparency in model testing. This will help to improve the credibility of complex models. These are often dismissed not only for being poorly communicated, but also because potential users do not obtain sufficient information to assess whether or not the model is good enough for its intended purpose. Post-hoc POM can help close this gap, and once more complex models have been tested and tuned in a similar way, less experimental, formal protocols for this approach are likely to emerge.

## Methods

### Procedure for Applying Pattern Testing

Since each of the four main pattern sets were derived from different studies it was necessary to define four separate ALMaSS scenarios to test each of the four sets. Using the fitting criteria described under POM Procedure above and iterative procedure was carried out. Parameter fitting and code modification was carried out by iterating comparisons to pattern set 1 (age structure and density). When an acceptable pattern set 1 fit was obtained, pattern set 2 (densities across multiple habitat types) was fitted, subsequently rechecking pattern set 1 fits. Once both pattern set 1 and pattern set 2 patterns were adequately replicated, pattern set 3 (dispersal patterns) was incorporated into the cycle, and finally pattern set 4 (the ability to create vole cycling). Due to the length of time taken to run a single replicate (between 30 minutes and 12 hours) the number of replicates and iterations of the model cycle needed to be kept to a minimum. Hence by necessity the precision of fit to each successive pattern set was relaxed to prevent an unfeasibly long fitting process, as well as over-fitting. The detailed procedure is described below:

#### Detailed parameter fitting procedure

This procedure is based on the experience with similar tests for other species modeled within the ALMaSS framework [44], [63]. The aim was to achieve a fit to the parameters with as few iterations as possible, whilst facilitating understanding of the model and the sensitivity analysis. The procedure used for this study was divided into 5 steps (1–5) each with a number of sub-steps and was as follows:

1. Pattern set 1 patterns
  1. Create a parameterization set and define a tolerance for fitting (in this case with a deviation of <2% for P.1–1 to P1.3, and simultaneously minimizing deviance from P1.4 & P1.5).
  2. Run the model varying each parameter in turn by e.g. ±5, 10 and 20% of its initial value. Run a minimum number of replicates of unique parameter set, usually 8–12.
  3. Create a set of diagrams describing the mean change in model outputs (pattern set 1 patterns) with changing parameter values (as Fig. 6, but with fewer points)
  4. Identify the next set of parameters which will reduce the mean across pattern deviation for the next iteration. This could be done in two ways, either using simple hill climbing, or by evaluating the responses on the charts and estimating which combination of parameter values should make a clear improvement to the fit. The latter approach requires comparison of responses across two or more parameters and then estimating the necessary change in all parameters to achieve the desired fit. Both methods were used depending on time constraints. Hill climbing was primarily used for fine tuning and when long periods of unattended model runs were possible, estimation worked best when the parameter values were far from optimum fit and rapid progress was needed.
  5. Iterate this cycle (go to 1.1) until all model outputs match their respective patterns within the given tolerance or until it is apparent that a fit is impossible. In the latter case this would manifest itself as the inability to find a combination of patterns that met the tolerance criteria.
  6. If a fit was possible go to 2, otherwise using charts from 1.3 and experience gained during the fitting to implement changes to the model structure, and return to 1.
2. Pattern set 2 patterns
  1. Test the parameterization set against pattern set 2 patterns. Tolerance was set as a mean deviation of <0.5 ln scale, and no individual values of >1.0 ln scale.
  2. If a fit could be obtained within acceptance limits using the set of parameter values fitted in ‘1’ above then parameter values were not altered, although the category into which habitats were classified could be altered to obtain better fits. Note it is possible to reach this point from 2.5 via 1.1–1.6
  3. If a fit was possible and any modifications to habitat quality categories had been carried out, then return to 1, if no modification and a fit obtained, then go to 3.1, otherwise got to 2.4.
  4. Alter parameter values until a fit was found or it was determined that a fit was impossible. In this case since there was only one type of pattern as a single metric to be assessed (i.e. the summed deviation from a perfect fit on natural log scale across all habitat season combinations for which there were patterns). If a fit was found go to 2.5, otherwise attempt code modification and return to 1.1.
  5. Return to 1.1 with the new parameterization to check that changes have not significantly altered the pattern set 1 fit, then if not, proceed to 3.1, otherwise iterate 1.1–2.5 each time restricting the parameter set for pattern set 1 to prevent repeating a previous pattern found not to fit both pattern sets.
3. Pattern set 3 patterns
  1. Test outputs against pattern set 3 patterns (see individual pattern set 3 criteria).
  2. If a fit is achieved then go to 4, otherwise modify parameters again to obtain a fit.*
  3. Return to 1 with the constraints that previous fits to pattern sets 1 & 2 were not allowed to be repeated to prevent endless iterations.
4. Pattern set 4 patterns
  1. Test outputs against pattern set 4 patterns (see individual pattern set 3 criteria).
  2. If a fit is achieved then go to 6, otherwise modify parameters again to obtain a fit.*
  3. Return to 1 with the constraints that previous fits to pattern set 1, 2 & 3 were not allowed.
5. Create an extended version of the charts in 1.3 as sensitivity plots of the final model configuration and parameterization.

*Program changes were not required for these steps in this study. This was serendipitous since there was no reason to expect this *a priori*.

Following parameterization, sensitivity analysis was carried out primarily with those patterns derived from pattern set 1 [47]. This restriction to pattern set 1 was for logistical reasons. The logistics of multi-dimension testing of 15 parameters at 11 values and four simulation types (each must be run separately for each parameter/value combination) was simply too overwhelming.

Following sensitivity analysis, the ODdox documentation was updated (see http://www2.dmu.dk/ALMaSS/ODDox/Field_Vole/V2_0/index.html), and reference folders containing executable files and input files needed for POM testing were archived at http://ccpforge.cse.rl.ac.uk/gf/project/almass/frs.

## Supporting Information

File S1 **Numeric real world data used for pattern sets 1–4.**
(XLSX)
